# Supplementary material for: Clinical Evaluation of the BIOFIRE SPOTFIRE Respiratory Panel
Source: Viruses. 2024 Apr 13;16(4):600. doi: 10.3390/v16040600 (PMC11055108; doi:10.3390/v16040600)
Supplement: Supplementary file 1 [file viruses-16-00600-s001.zip › viruses-2909930-supplementary.pdf]

Table S1 Patient demographics

		Number (%)
**NIH style age groups**	Neonates or newborns (birth to <1 month)	1 (0.68%)
	Infants (1 month to <1 year)	5 (3.42%)
	Children (1 year to 12 years)	89 (60.96%)
	Adolescents (13 years to 17 years)	7 (4.79%)
	Adults (18 years to 64 years)	25 (17.12%)
	Older adults (65 years or older)	19 (13.01%)
**Gender**	Male	82 (56.16%)
	Female	64 (43.84%)
**Patient type**	Inpatients	76 (52.05%)
	Outpatients	70 (47.95%)

Table S2 Frequency of detection for individual pathogens and discrepant analysis (upper respiratory tract specimens)

Pathogen targets	No. of positive specimens		Discrepant analysis
	RP2.1*plus*	SPOTFIRE R	
Adenovirus	15	18	Two specimens were positive by RP2.1*plus* and negative by SPOTFIRE R Panel. The Ct values were 40 by the alternative method.<br>Two specimens were negative by RP2.1*plus* and positive by SPOTFIRE R Panel. The Ct values were 37.8 and 40 by the alternative method.<br>Three specimens were negative by RP2.1*plus* and positive by SPOTFIRE R Panel. The results were negative by the alternative method. The patients had no recent history of adenovirus infection.<br>**SPOTFIRE R Panel: 2FN, 3FP, 113TN and 15TP**
**Coronavirus**<br>229E<br>HKU1<br>NL63<br>OC43<br>Unclassified	 3<br>2<br>7<br>6<br>0	 3<br>2<br>7<br>6<br>1	Reported as 'coronavirus (seasonal)' without subtype by SPOTFIRE R Panel.<br>One specimen was negative by RP2.1*plus* and positive by SPOTFIRE R Panel. The result was negative by the alternative method. The patient had no recent history of coronavirus infection.<br>**SPOTFIRE R Panel: 0FN, 1FP, 114TN and 18TP**
SARS-CoV-2	17	18	One specimen was positive by RP2.1*plus* and negative by SPOTFIRE R Panel. The result was negative by the alternative method. The patient had no recent history of SARS-CoV-2 infection.<br>Two specimens were negative by RP2.1*plus* and positive by SPOTFIRE R Panel. The Ct values were 40 and 44.3 by the alternative method.<br>**SPOTFIRE R Panel: 0FN, 0FP, 118TN and 15TP**
Metapneumovirus	6	6	**SPOTFIRE R Panel: 0FN, 0FP, 127TN and 6TP**
Rhinovirus/ enterovirus	23	34	One specimen was positive by RP2.1*plus* and negative by SPOTFIRE R Panel. Rhinovirus was detected by the alternative method.<br>Eleven specimens were negative by RP2.1*plus* and positive by SPOTFIRE R Panel. Rhinovirus was detected by the alternative method.<br>One specimen was negative by RP2.1*plus* and positive by SPOTFIRE R Panel. Rhinovirus and enterovirus were not detected by the alternative methods. No clinical history was available.<br>**SPOTFIRE R Panel: 1FN, 1FP, 98TN and 33TP**
**Influenza A virus**	**41**	**41**	**SPOTFIRE R Panel: 0FN, 0FP, 92TN and 41TP**
No subtype/ equivocal	1	1	H1-2009 was detected by the alternative method, The Ct value was 30.65.
H1-2009	14	13<br>(+ 1 no subtype)	The result of SPOTFIRE R Panel for the discrepant specimen was 'no subtype'. The Ct value was 36.2 by the alternative method.<br>**SPOTFIRE R Panel: 1FN, 0FP, 119TN and 13TP**
H3	25<br>(+1 no subtype)	26	The result of RP2.1*plus* for the discrepant specimen was 'no subtype' in the first run and 'equivocal' in the repeated run. For SPOTFIRE R Panel, the result was 'H3'. The Ct value was 31.6 by the alternative method.<br>**SPOTFIRE R Panel: 0FN, 0FP, 106TN and 27TP**
Influenza B virus	8	8	**SPOTFIRE R Panel: 0FN, 0FP, 125TN and 8TP**
**Parainfluenza virus**<br>Type 1<br>Type 2<br>Type 3<br>Type 4	 7<br>5<br>7<br>3	 7<br>5<br>7<br>4	Reported as 'parainfluenza virus' without subtype by SPOTFIRE R Panel.<br>The discrepant specimen was negative by RP2.1*plus* and positive by SPOTFIRE R Panel. Parainfluenza virus 4 was detected by the alternative method.<br>**SPOTFIRE R Panel: 0FN, 0FP, 110TN and 23TP**
Respiratory syncytial virus	10	10	One specimen was positive by RP2.1*plus* and negative by SPOTFIRE R Panel. The result was negative by the alternative method, and vice versa.<br>**SPOTFIRE R Panel: 0FN, 1FP, 123TN and 9TP**
B. parapertussis	3	4	One specimen was negative by RP2.1*plus* and positive by SPOTFIRE R Panel. The result was negative by the alternative method. No clinical history was available.<br>**SPOTFIRE R Panel: 0FN, 1FP, 129TN and 3TP**
B. pertussis	2	2	**SPOTFIRE R Panel: 0FN, 0FP, 131TN and 2TP**
C. pneumoniae	1	1	**SPOTFIRE R Panel: 0FN, 0FP, 132TN and 1TP**
M. pneumoniae	13	13	**SPOTFIRE R Panel: 0FN, 0FP, 120TN and 13TP**

Ct, threshold cycle; FN, false negative; FP, false positive; TN, true negative; TP, true positive

Table S3 Discrepant analysis of co-detections (upper respiratory tract specimens)

Specimen		Results			Remarks
**No.**	**Type**	**RP2.1*plus***	**SPOTFIRE R**	**Discrepant analysis**	
1	NPS	Flu A H3	Flu A H3<br>RV/EV	Rhinovirus was detected	RP2.1*plus* < SPOTFIRE R
2	NPS	Flu A H3<br>RV/EV	Flu A H3<br>RV/EV<br>BPP	*B. parapertussis* was not detected. No clinical history was available.	RP2.1*plus* = SPOTFIRE R
9	NPS	Flu A H3	Flu A H3<br>ADV<br>PIV	Adenovirus was not detected. No recent history of adenovirus infection.<br>Parainfluenza virus 4 was detected	RP2.1*plus* < SPOTFIRE R
10	NPS	Flu A H3	Flu A H3<br>RV/EV	Rhinovirus was detected	RP2.1*plus* < SPOTFIRE R
19	NS-TS	SARS-CoV-2<br>ADV	ADV	SARS-CoV-2 was not detected. No recent history of SARS-CoV-2 infection.	RP2.1*plus* = SPOTFIRE R
20	NPS	MP	MP<br>RV/EV	Rhinovirus was detected. Rhinovirus was detected by RP2.1*plus* 5 days ago.	RP2.1*plus* < SPOTFIRE R
22	NS	PIV4	PIV4<br>ADV	Adenovirus was not detected. No recent history of adenovirus infection.	RP2.1*plus* = SPOTFIRE R
28	NPS	ADV<br>RV/EV	RV/EV	Adenovirus was detected (Ct value: 40)	RP2.1*plus* > SPOTFIRE R
29	NPS-TS	ADV	ADV<br>CoV<br>RV/EV	Seasonal coronavirus was not detected with no recent history of infection<br>Rhinovirus was detected	RP2.1*plus* < SPOTFIRE R
32	NPS	PIV3<br>CoV OC43	PIV<br>CoV<br>ADV<br>SARS-CoV-2	Adenovirus was not detected with no recent history of infection<br>SARS-CoV-2 was detected (Ct value: 44.3)	RP2.1*plus* < SPOTFIRE R
41	NPS	PIV3<br>RV/EV	PIV	Rhinovirus was detected	RP2.1*plus* > SPOTFIRE R
45	NPS	RSV<br>CoV NL63	RSV<br>CoV<br>RV/EV	Rhinovirus was detected	RP2.1*plus* < SPOTFIRE R
55	NPS	Flu A H1-2009	Flu A H1-2009<br>RV/EV	Rhinovirus was detected	RP2.1*plus* < SPOTFIRE R
70	NPS	RV/EV<br>MP	RV/EV<br>MP<br>ADV<br>SARS-CoV-2<br>RSV	Adenovirus was detected (Ct value: 40)<br>SARS-CoV-2 was detected (Ct value: 38.2)<br>RSV was not detected. No clinical history was available.	RP2.1*plus* < SPOTFIRE R
78	NPS	MP	MP<br>ADV	Adenovirus was detected (Ct value: 37.8)	RP2.1*plus* < SPOTFIRE R
79	NPS	BP	BP<br>RV/EV	Rhinovirus or enterovirus was not detected. No clinical history was available.	RP2.1*plus* = SPOTFIRE R
84	NPS	Flu A H1-2009	Flu A H1-2009<br>RV/EV	Rhinovirus was detected	RP2.1*plus* < SPOTFIRE R
87	NPS	PIV2	PIV<br>RV/EV	Rhinovirus was detected	RP2.1*plus* < SPOTFIRE R
91	NPS	SARS-CoV-2<br>RSV	SARS-CoV-2<br>RSV<br>RV/EV	Rhinovirus was detected	RP2.1*plus* < SPOTFIRE R
102	NPS	ADV<br>RSV	ADV	Respiratory syncytial virus was not detected	RP2.1*plus* = SPOTFIRE R
126	NPS	CoV NL63	CoV<br>RV/EV	Rhinovirus was detected	RP2.1*plus* < SPOTFIRE R
137	NPS-TS	CoV NL63<br>PIV2 & 4	CoV<br>PIV<br>RV/EV	Rhinovirus was detected	RP2.1*plus* < SPOTFIRE R
142	NPS	ADV<br>RV/EV	RV/EV	Adenovirus was detected (Ct value: 40)	RP2.1*plus* > SPOTFIRE R

ADV, adenovirus; BPP, *Bordetella parapertussis*; CoV, coronavirus; Ct, threshold cycle; Flu, influenza; MP, *Mycoplasma pneumoniae*; NPS, nasopharyngeal swab; NS, nasal swab; TS, throat swab; PIV, parainfluenza virus; RSV, respiratory syncytial virus; RV/EV, rhinovirus/enterovirus

Table S4 Results on lower respiratory tract specimens

Specimen No.	Type	RP2.1*plus* results	SPOTFIRE R results	Remarks
63	Sputum	1st run: flu A (no subtype)<br>2nd run: flu A (no subtype)	Flu A (no subtype)	Alternative method:<br>H1-2009, Ct value: 41
83	BALF	*Chlamydia pneumoniae*	*Chlamydia pneumoniae*	
90	Sputum	SARS-CoV-2<br>Flu A H3	SARS-CoV-2<br>Flu A H3	
100	Sputum	Negative	Negative	
101	Sputum	Parainfluenza virus 3	Parainfluenza virus	
103	Sputum	Negative	Negative	
114	Sputum	Respiratory syncytial virus	Respiratory syncytial virus	
115	ETA	Negative	Negative	
116	Sputum	Flu A H3	Flu A H3	
117	Sputum	Negative	Negative	
118	Sputum	SARS-CoV-2	SARS-CoV-2	
119	Sputum	SARS-CoV-2	SARS-CoV-2	
120	Sputum	Negative	Negative	

BALF, bronchoalveolar lavage fluid; ETA, endotracheal aspirate; flu A, influenza A virus; SARS-CoV-2, severe acute respiratory syndrome coronavirus 2.

Table S5 Results on EQAP samples

Sample	RP2.1*plus* results	SPOTFIRE R results	Expected results
1	Influenza B virus<br>Parainfluenza virus 1	Influenza B virus<br>Parainfluenza virus	Influenza B virus<br>Parainfluenza virus 1
2	Influenza A virus H1-2009	Influenza A virus H1-2009	Influenza A virus H1-2009
3	Influenza B virus<br>Rhinovirus/enterovirus	Influenza B virus<br>Rhinovirus/enterovirus	Influenza B virus<br>Rhinovirus/enterovirus
4	Influenza A virus H3<br>Parainfluenza virus 4	Influenza A virus H3<br>Parainfluenza virus	Influenza A virus H3<br>Parainfluenza virus 4
5	Adenovirus<br>*Bordetella pertussis*	Adenovirus<br>*Bordetella pertussis*	Adenovirus<br>*Bordetella pertussis*
